# Efficacy of ATR inhibitors as single agents in Ewing sarcoma

**DOI:** 10.18632/oncotarget.11643

**Published:** 2016-08-26

**Authors:** Maria Nieto-Soler, Isabel Morgado-Palacin, Vanesa Lafarga, Emilio Lecona, Matilde Murga, Elsa Callen, Daniel Azorin, Javier Alonso, Andres J. Lopez, Andre Nussenzweig, Oscar Fernandez-Capetillo

**Affiliations:** ^1^ Genomic Instability Group, Spanish National Cancer Research Centre (CNIO), Madrid, Spain; ^2^ Laboratory of Genome Integrity, National Cancer Institute, NIH, Bethesda, Maryland, USA; ^3^ Department of Pathology, Hospital Universitario Niño Jesus, Madrid, Spain; ^4^ Pediatric Solid Tumor Laboratory, Institute of Rare Disease Research, ISCIII, Madrid, Spain; ^5^ Center for Chromosome Stability, Department of Cellular and Molecular Medicine, Panum Institute, University of Copenhagen, Denmark; ^6^ Science for Life Laboratory, Division of Translational Medicine and Chemical Biology, Department of Medical Biochemistry and Biophysics, Karolinska Institute, Stockholm, Sweden

**Keywords:** ATR, Ewing sarcoma, replication stress, DNA repair, cancer

## Abstract

Ewing sarcomas (ES) are pediatric bone tumors that arise from a driver translocation, most frequently EWS/FLI1. Current ES treatment involves DNA damaging agents, yet the basis for the sensitivity to these therapies remains unknown. Oncogene-induced replication stress (RS) is a known source of endogenous DNA damage in cancer, which is suppressed by ATR and CHK1 kinases. We here show that ES suffer from high endogenous levels of RS, rendering them particularly dependent on the ATR pathway. Accordingly, two independent ATR inhibitors show *in vitro* toxicity in ES cell lines as well as *in vivo* efficacy in ES xenografts as single agents. Expression of EWS/FLI1 or EWS/ERG oncogenic translocations sensitizes non-ES cells to ATR inhibitors. Our data shed light onto the sensitivity of ES to genotoxic agents, and identify ATR inhibitors as a potential therapy for Ewing Sarcomas.

## INTRODUCTION

Genomic instability is widespread in cancer cells, as already noticed in the Boveri studies of the early 20^th^ century [1]. Hence, targeting genomic instability offers an opportunity to develop treatments that preferentially kill cancer cells. This idea gained momentum with the development of therapies such as inhibitors of poly (ADP-ribose) polymerases (PARPs), which are highly toxic for cells with mutations in *BRCA1/2* and thus defective in DNA repair by Homologous Recombination (HR) [2, 3]. An alternative to targeting a specific mutation is to exploit the presence of high endogenous levels of DNA damage in tumors. A well-established source of genomic instability in cancer is oncogene-induced RS [4]. As a consequence, targeting RS-response kinases ATR and CHK1 is preferentially toxic for tumors experiencing high levels of RS such as MYC-induced lymphomas, MLL-translocation driven leukemias or H-RAS driven fibrosarcomas [5-7]. In this context, the identification of cancers presenting high levels of RS is important to guide the use of ATR and CHK1 inhibitors in cancer therapy [8].

Several reasons led us to hypothesize that Ewing Sarcomas (ES) might be suffering from RS. First, the EWS/FLI1 translocation product is a *bona fide* oncogene due to its capacity to transform mouse fibroblasts [9] and, as mentioned, oncogenes are a known source of RS [4]. Second, current ES treatments use chemicals that perturb DNA replication such as the alkylating agent temozolomide or topoisomerase I inhibitors. Third, EWSR1 interacts with BARD1 which, together with BRCA1, regulates recombination processes that are essential for DNA replication [10]. Moreover, and similar to *BRCA* mutant tumors [2, 3], ES are also sensitive to PARP inhibitors [11, 12]. Finally, EWSR1-deficient mice present DNA damage, anemia and skeletal abnormalities [13, 14]; which are also found in mice with reduced ATR levels that accumulate substantial amounts of RS [15]. For these reasons, we explored whether ES indeed suffer from high levels of RS and whether this would render them sensitive to ATR inhibition.

## RESULTS

The presence of high levels of RS in cancer cells creates a pressure to acquire mutations that suppress RS and therefore facilitate their growth [16-18]. Supporting this view, CHK1 overexpression increases the efficiency of transformation by RAS, by suppressing oncogene-induced RS [19, 20]. In addition, increased *CHEK1* expression and/or gene copy number gains have been observed in tumors with a high degree of genomic instability, which correlated with an increased sensitivity to ATR or CHK1 inhibition [21, 22]. We therefore reasoned that the presence of high CHK1 levels could be used to identify tumor types with elevated amounts of RS.

**Figure 1:**
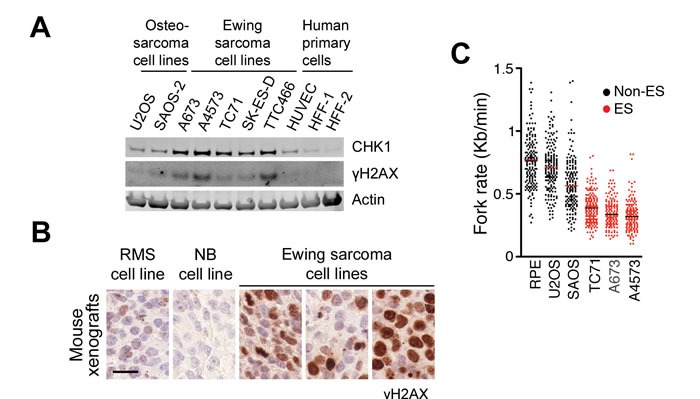
Increased RS levels in Ewing sarcomas **A**. CHK1 and γH2AX levels evaluated by WB on several ES lines, together with 2 osteosarcoma lines and 3 human primary cell types. **B**. γH2AX IHC on mouse xenografts from 3 ES lines (A4573, A673 and TC71), and two independent xenografts from ES-related tumors (rhabdomyosarcoma (RMS); neuroblastoma (NB)). Scale bar (black) indicates 20 μm. **C**. Fork rates were measured in stretched DNA fibers prepared from non-ES (RPE, U2OS, SAOS) and ES (TC71, A673 and A4573) cell lines. At least 200 tracks were measured per condition. ***P<0.001 by two-tailed *t* test.

To explore this possibility, we first interrogated the human Cancer Cell Line Enciclopedia (CCLE) dataset for *CHEK1* mRNA expression (https://portals.broadinstitute.org/ccle/home) [23]. Supporting our view, *CHEK1* levels are highest in all kinds of hematopoietic tumors, where ATR and CHK1 inhibitors are particularly effective [5, 6, 22, 24]. After mesothelioma, ES were the solid tumors showing the highest levels of *CHEK1* mRNA from the CCLE dataset. In agreement with this, CHK1 protein levels were distinctively higher in a panel of ES lines than in primary cells or other osteosarcomas (Figure 1A). The presence of high CHK1 levels correlated with an increased phosphorylation of histone H2AX (γH2AX) in ES cell lines, supporting the presence of RS in these cells. Immunohistochemistry (IHC) of ES xenografts confirmed the presence of cells positive for γH2AX, which was more abundant than on xenografts from other related tumors such as neuroblastoma or rhabdomyosarcoma (Figure 1B). Moreover, γH2AX showed a pan-nuclear distribution, which is the pattern that is found in tumors with high levels of RS [5] and induced by ATR or CHK1 inhibitors [25, 26]. Finally, and to directly evaluate DNA replication in ES cells, we analyzed replication fork progression on isolated stretched DNA fibers. These experiments revealed that fork progression is slower on any ES line tested (TC71, A673 and A4573) than in human primary retinal pigmentum epithelial (RPE) cells or in U2OS and SAOS osteosarcoma cell lines (Figure 1C). Collectively, these data reveal the presence of RS in Ewing sarcomas suggesting that these tumors could be particularly responsive to ATR inhibitors.

**Figure 2:**
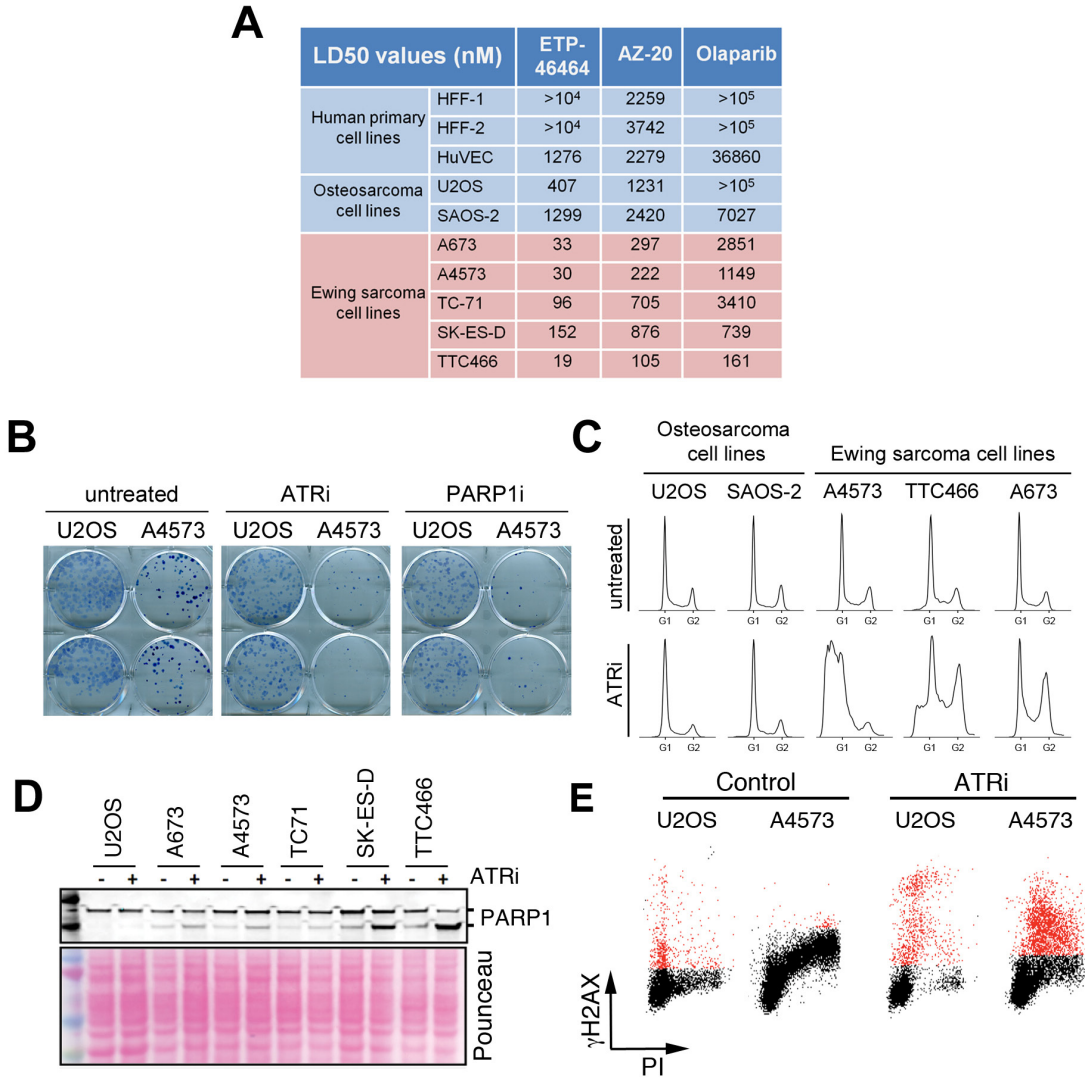
Sensitivity of ES to ATR inhibitors *in vitro* **A.** LD50 values of 2 independent inhibitors (ETP-46464 [26] and AZ20 [27]) and a PARP1 inhibitor (olaparib, PARPi hereafter) on the same lines used in Figure 1B. The LD50 values for temozolomide, currently used in ES chemotherapy, were above 100 μM in all lines tested. **B.** Clonogenic assays illustrating the differential effects of ATRi and PARPi on U2OS and A4573 cells. **C.** DNA content was assessed by flow cytometry on 2 non-ES osteosarcoma lines and 3 ES lines exposed to ATRi for 72 hrs (1 μm). **D.** Western blot illustrating the cleavage of PARP1 on ES lines and U2OS upon a short exposure to ATRi (1 μM, 4 hrs). **E.** FACS analysis of DNA content (PI) and H2AX phosphorylation in U2OS and A4573 cells exposed to ATRi (10 μM, 5 hrs), illustrating the increased levels of ATRi-induced RS (as measured by γH2AX in cells with an S-phase DNA content) in ES cells.

To determine the efficacy of ATR inhibitors on ES, we first calculated the lethal dose 50 (LD50) of these compounds *in vitro* (Figure 2A). Two independent ATR inhibitors (ETP-46464: ATRi hereafter [26] and AZ20 [27]) showed higher toxicity for ES cells than for human primary cells or non-ES osteosarcomas, and significantly lower LD50 values than the PARP inhibitor olaparib. Moreover, the toxicity of ATR inhibitors correlated with the levels of CHK1 and γH2AX present on ES lines (see Figure 1A), consistent with the toxicity of these compounds being proportional to the levels of RS. Noteworthy, one of the cell lines from our panel was U2OS, a non-ES osteosarcoma cell line recently identified as being highly sensitive to ATR inhibitors due to its reliance on the ALT pathway for telomere maintenance [28]. The toxicity of ATR inhibitors on all ES lines tested was higher (up to 20-fold) than on U2OS. Clonogenic assays confirmed a greater impact of ATR inhibition on ES cells than on U2OS (Figure 2B). Together, these results support that ATR inhibitors are especially toxic for ES cells.

Next, we analyzed the effects of ATR inhibition in ES cells. First, flow cytometry analyses of DNA content confirmed an increased toxicity of ATRi in ES lines, at doses at which no obvious impact of the inhibitor was observed on the cell cycle distribution of U2OS or SAOS-2 osteosarcoma cells (Figure 2C). The compound triggered apoptosis in ES cells, evidenced by the emergence of cells with a subG1 DNA content, as well as by the caspase-mediated cleavage of PARP1 (Figure 2D). Besides apoptosis, the main mechanism by which ATR inhibitors kill cells is by forcing premature mitotic entry in cells suffering from RS [29]. Accordingly, in ES cell lines ATR inhibition led to the accumulation of cells in the S/G2 phases of the cell cycle. Moreover, flow cytometry analyses of H2AX phosphorylation together with DNA content revealed that ATR inhibition led to increased γH2AX levels specifically in S phase, and which were exacerbated in ES (A4573) cells compared to U2OS (Figure 2E). Thus, the sensitivity of ES to ATR inhibition correlates with an increased induction of RS by the compound in these cells.

**Figure 3:**
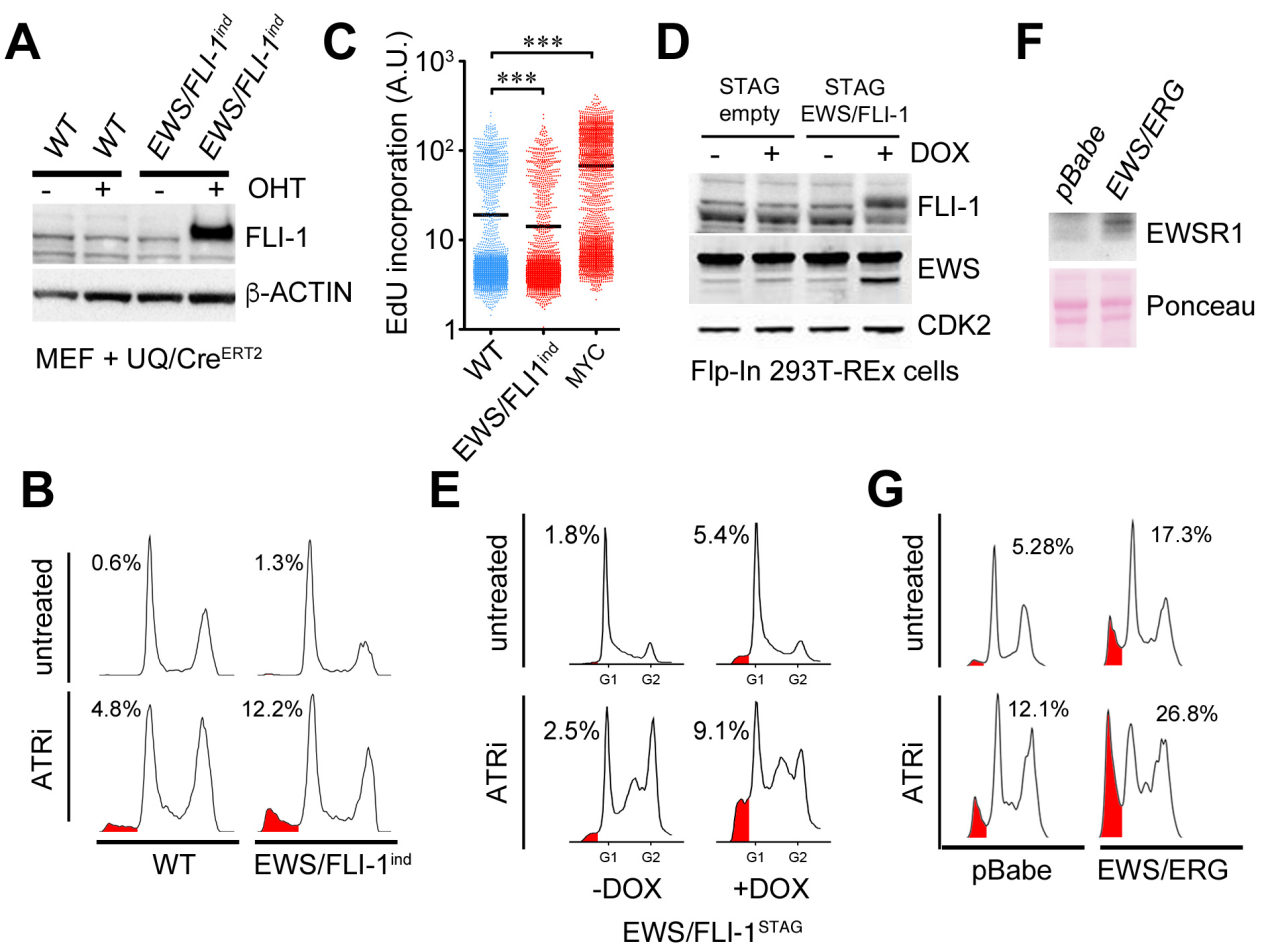
Expression of EWSR1 translocations sensitizes cells to ATRi **A.** WB illustrating the expression of EWS/FLI1 (measured with a FLI1 antibody) that can be obtained in EWS/FLI1^ind^ MEF upon 4-hydroxy-tamoxifen (OHT)-induced activation of a Cre-ERT2 expressed from the ubiquitin promoter (UQ/Cre^ERT2^) [41]. OHT was added for 48 hrs at 1 μM. β-ACTIN was used as loading control. **B.** DNA content analyses by flow cytometry illustrating the toxicity of ATRi (5μM, 48 hrs) on WT and EWS/FLI1^ind^ MEF harboring UQ-Cre^ERT2^ exposed to OHT (1 μM, 48 hrs). SubG1 populations are shaded in red and their percentages are indicated. **C.** DNA replication rates of WT and EWS/FLI1^ind^ MEF harboring UQ-Cre^ERT2^ exposed to OHT, as well as of WT MEF infected with a retrovirus expressing the MYC oncogene were evaluated by quantifying the incorporation of EdU per nucleus by High Throghput Microscopy. **D.** WB illustrating the expression of EWS/FLI1 (measured with EWS and FLI-1 antibodies) that can be obtained in Flip-In 293T-Rex cells carrying a STAG-EWS/FLI1 cDNA (EWS/FLI1^STAG^) upon induction with doxycycline (Dox) (200 ng/ml, 48 hrs). The levels in a clone of Flip-In 293T-Rex cells expressing only the STAG peptide are shown as expression controls. CDK2 was used as loading control. **E.** DNA content analyses by flow cytometry illustrating the toxicity of ATRi (1μM, 24 hrs) on EWS/FLI1^STAG^ cells exposed or not to Dox (48 hrs). SubG1 populations are shaded in red and their percentages are indicated. **F.** WB illustrating the expression of EWS/ERG (measured with an EWSR1 antibody) that can be obtained in MEF upon infection with a EWS/ERG expressing retrovirus (or empty vector; pBabe). β-ACTIN was used as loading control. **G.** Flow cytometry illustrating the toxicity of ATRi (5μM, 48 hrs) on MEF infected with an EWS/ERG expressing retrovirus (or empty vector). SubG1 populations are shaded in red and their percentages are indicated.

Next, and to determine whether the sensitivity towards ATR inhibitors observed on ES cells was not something particular of the chosen cell lines but rather a consequence of the initiating oncogenic translocation, we first used a mouse transgenic line where EWS/FLI1 expression can be induced by the Cre recombinase (EWS/FLI1^ind^) [30] (Figure 3A). Cre expression was sufficient to sensitize EWS/FLI1^ind^ MEFs to ATR inhibition (Figure 3B). Interestingly, and in contrast to other oncogenes that sensitize to limited ATR activity such as MYC [5], EWS/FLI expression did not increase DNA replication rates as measured by EdU incorporation (Figure 3C). To test the effect of EWS/FLI1 expression in human cells and independently of Cre we generated a doxycycline-inducible EWS/FLI1 expressing line in human Flip-In T-Rex 293T cells (293^EWS/FLI1^) (Figure 3D). Similar to the observations in EWS/FLI1^ind^ MEFs, doxycyclin exposure sensitized 293^EWS/FLI1^ cells to ATRi (Figure 3D, 3E). In addition to EWS/FLI1, expression of EWS/ERG also sensitized cells to ATR inhibition (Figure 3F, 3G). In fact, one of the ATRi-sensitive ES cell lines tested above (TTC466) carries an EWS/ERG translocation instead of EWS/FLI1. In summary, expression of EWSR1 involving translocations sensitizes human and mouse cells to ATR inhibitors.

**Figure 4:**
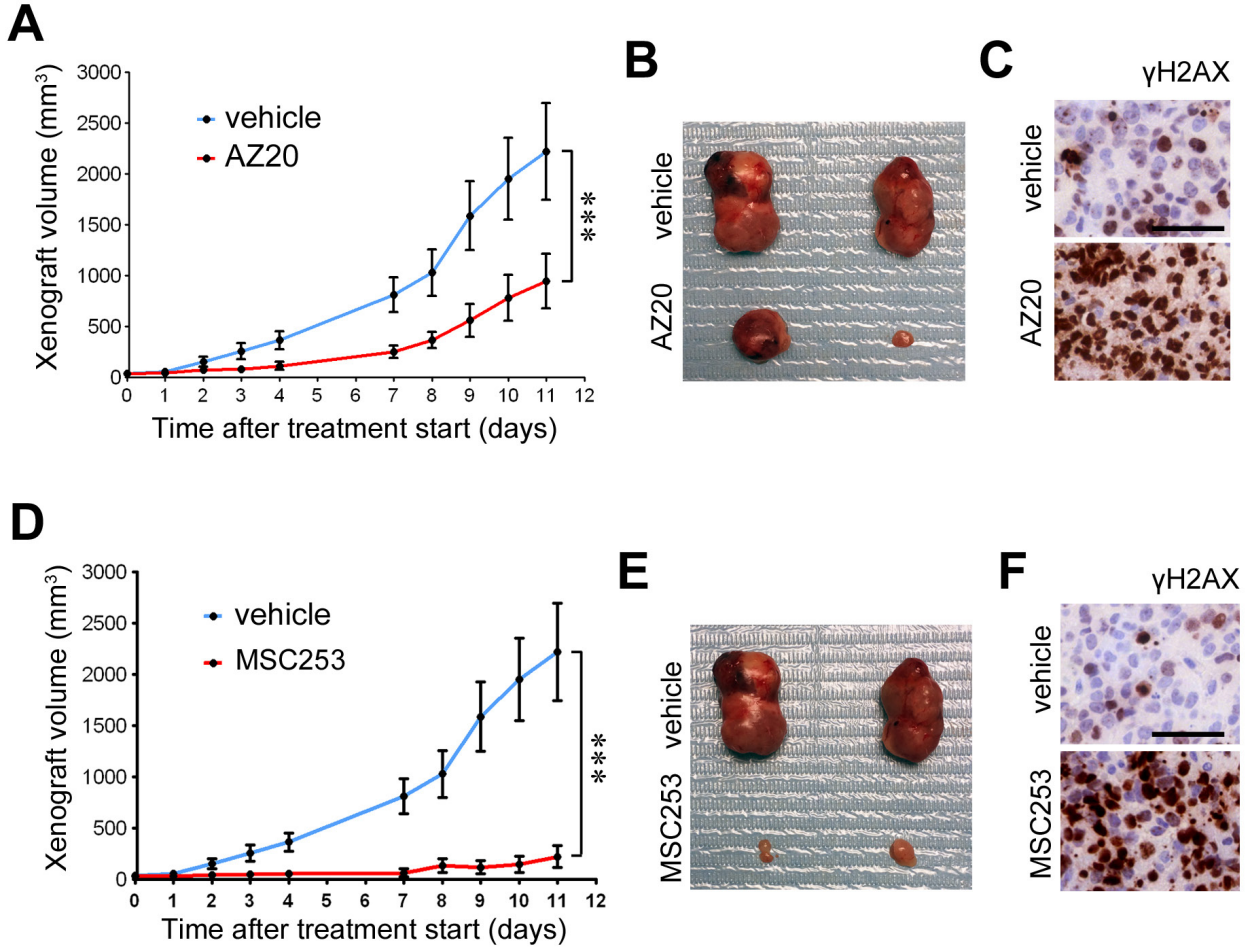
Efficacy of ATR inhibitors in ES xenografts as single agents **A.** Efficacy of AZ20 as monotherapy on the growth of ES xenografts (A4573). Treatment started when tumors became palpable. **B.** Examples of the tumor sizes observed at endpoint from A. **C.** γH2AX IHC on xenografts from A 48 hrs after starting the treatment. Scale bar indicates 30 μm. **D.** Efficacy of an independent ATR inhibitor (MSC253) as monotherapy on the growth of ES xenografts (A4573). Treatment started when tumors became palpable. **E.** Examples of the tumor sizes observed at endpoint from D. **F.** γH2AX IHC on xenografts from D 48 hrs after starting the treatment. Scale bar indicates 30 μm. Error bars indicate s.d. ***P < 0.001.

Finally, to determine the efficacy of ATR inhibitors *in vivo*, we evaluated their antitumoral effects using xenografts in immunodeficient mice (SCID). Remarkably, oral administration of two independent ATR inhibitors reduced the growth of xenografts from A4573 ES cells (Figure 4). Moreover, xenografts from mice treated with ATR inhibitors presented a generalized accumulation of cells with pan-nuclear γH2AX, consistent with the mechanism of action of ATR and CHK1 inhibitors [5, 22] (Figure 4C,4F). Of note, and whereas all current clinical trials using ATR inhibitors rely on combination therapies with additional genotoxic agents (https://clinicaltrials.gov/), both ATR inhibitors were used as single agents in these experiments.

## DISCUSSION

Metastatic Ewing sarcoma is a pediatric tumor of very poor prognosis, due to the lack of efficient therapies. Current treatments involve genotoxic agents such as temozolomide or irinotecan, whose mechanism of action involves the generation of RS. In what regards to new alternatives, ES cells were also reportedly sensitive to PARP inhibitors [23, 31]. However, initial clinical trials failed to see a response to these compounds in ES patients [32], and thus new therapies are still needed. We here provide evidence of a distinctively high sensitivity of Ewing sarcomas to ATR inhibitors, which correlates with high levels of endogenous RS in these tumors. Both ATR inhibitors were significantly more toxic than the PARP inhibitor olaparib in all ES lines tested. In addition, all ES cell lines were more sensitive to ATR inhibition than the ALT-positive cell line U2OS, recently reported as highly sensitive to these agents [28].

Our discovery of high endogenous levels of RS in ES cells also helps to explain the intrinsic sensitivity of ES to agents that perturb DNA replication. As observed in other tumors suffering from RS, such as recombination-deficient ovarian cancers [21] or MYCN-driven neuroblastomas [33], ES cells present high levels of CHK1 expression, which helps them deal with the presence of RS. As a consequence, these tumors become addicted to a proficient ATR/CHK1 pathway for their survival, explaining the high sensitivity of ES cells to ATR inhibition. At this point, we do not know why the expression of EWSR1 translocation products drives RS in ES cells, yet since EWS/FLI1 is a transforming oncogene [9] it could simply be another case of oncogene-induced RS. However, and in contrast to other oncogenes such as MYC or RAS, EWS/FLI1 expression does not increase DNA replication, so that a novel mechanism must be in place to explain this synthetic lethal interaction. One interesting possibility is that the expression of EWSR1 fusions could perturb the function of endogenous EWSR1, which could be the source of RS and genomic instability of these tumors. Consistently, a recent report revealed a critical role of EWSR1 in facilitating the recruitment of DNA repair factors to sites of DNA damage [34]. In addition, previous work revealed that depletion of EWSR1 reduced the levels of several DNA damage response factors, due to alterations in alternative splicing [35]. Finally, and as mentioned before, the phenotypes of EWSR1-deficient mice are reminiscent of those found in ATR mutant mice [13-15]. Hence, it is possible that EWSR1 translocations could exert a dominant negative function over endogenous EWSR1, leading to RS and genomic instability in Ewing sarcomas. Regardless of how EWSR1 fusions generate RS, our work provides a basis to understand the sensitivity of ES to RS-inducing agents, and identifies ATR inhibitors as a potential therapy for ES.

## MATERIALS AND METHODS

### Cell culture

All human cell lines used in this study were cultured in RPMI (EuroClone) supplemented with 10% FBS (Lonza). Non-ES lines were acquired from ATCC. All ES lines were kindly provided by Dr. Enrique de Alava (IBiS, Spain). MEF from E13.5 d.p.c. embryos were generated by standard methods and grown in DMEM supplemented with 15% FBS and under normoxic conditions to minimize exposure to reactive oxygen species. For all experiments, MEF were used at a low passage (<3). For clonogenic assays, 500 cells were seeded per well on six-well plates and drugs were added 24 hr later. After 10 days, cells were fixed and stained with methylene blue at 0.33% (w/v) in methanol, subsequently washed in water and air-dried. For the calculation of LD50 values an II-XTT Cell Viability assay (Roche) was used.

### Xenografts

8-10 week-old CB17/lcr-Prkdc scid/Crl male mice were used. One million A4573 cells were inoculated subcutaneously in the right flank of mice. Growing tumor masses were measured with the aid of a Vernier caliper, and tumor volumes were calculated using the formula: (width x (length)^2^)/2. When tumor volumes reached about 100 mm^3^, mice were randomized into two equal groups and treatment started. 7 mice were used per treatment group. ATR inhibitors or vehicle were administered via oral gavage 5 times per week at a dose of 50 mg/Kg. MSC253 (kind gift from Merck KGaA, Darmstadt, Germany) was dissolved in 10%NMP (443778; Sigma-Aldrich), 50% PEG-300 (202371; Sigma-Aldrich) and 40% H2O. AZ20 has been previously described [27] and was dissolved in 10%NMP and 90% PEG-300. All mouse work was performed in accordance with the Guidelines for Humane Endpoints for Animals Used in Biomedical Research, and under the supervision of the Ethics Committee for Animal Research of the “Instituto de Salud Carlos III”.

### Immunoblotting and immunofluorescence

For protein extracts, cells were washed once with PBS, and lysed in RIPA buffer (Tris-HCl 50 mM, pH 7.4, NP-40 1%, Na-deoxycholate 0.25%, NaCl: 150 mM, EDTA 1 mM) containing protease and phosphatase inhibitors (Sigma) or in a buffer containing 50 mM Tris (pH 7.5), 8 M urea, and 1% 3-[(3-cholamidopropyl)-dimethylammonio]-1-propanesulfonate (CHAPS). Samples were resolved by SDS-PAGE and analyzed by standard Western blotting techniques. For immunofluorescence, cells were fixed with 4% PFA and permeabilized with 0.1% Triton-X100. Antibodies against EWSR1 (sc-6533 and sc-28327, Santa Cruz), FLI1 (sc-356, Santa Cruz), CHK1 (NCL, Novocastra), PARP1 (9542S, Cell Signaling), γH2AX (05-636, Millipore), β-ACTIN (A5316, Sigma) and CDK2 (sc-163, Santa Cruz) were used. Protein blot analyses were performed on the LICOR platform (Biosciences).

### Inmunohistochemistry

Tissues were fixed in formalin and embedded in paraffin for subsequent processing. 2.5-μM sections were treated with citrate for antigenic recovery and processed for immunohistochemistry with γH2AX (05-636, Millipore) antibody. Slides were scanned and digitalized with a MIRAX system (Zeiss) for further analysis.

### Flow cytometry

To measure viability, cells were collected, washed once with PBS, stained in a DAPI solution (0.2 μg/mL DAPI in PBS) and analysed by flow cytometry in a FACS Canto II (Becton-Dickinson) machine. For cell cycle profiles, cells were collected, washed with PBS and fixed in suspension in ice-cold 70% (v/v) ethanol in PBS. After washing in PBS, cells were stained in a PBS solution containing propidium iodide (10 μg/ml) and RNase A (0.5 mg/ml) and collected in a Becton-Dickinson FACS Calibur machine. For DNA content and γH2AX analysis, p-Ser139 H2AX (Millipore) antibodies were used as previously described [29]. Data was analyzed by using FACS Diva (BD Biosciences) and FlowJo (Treestar) softwares.

DNA fiber analyses Cells were pulse-labeled with 50 μM CldU (20 min) followed by 250 μM IdU (20 min). Labeled cells were collected and DNA fibers were spread in buffer containing 0.5% SDS, 200 mM Tris pH 7.4 and 50 mM EDTA. For immunodetection of labeled tracks, fibers were incubated with primary antibodies (for CldU, rat anti-BrdU; for IdU, mouse anti-BrdU) for 1 hour at room temperature and developed with the corresponding secondary antibodies for 30 minutes at room temperature. Mouse anti-ssDNA antibody was used to assess fiber integrity. Slides were examined with a Leica DM6000 B microscope, as described previously [36]. The conversion factor used was 1 μm=2.59 kb [37].

### Statistical analyses

Data were represented using Prism 5.0 (GraphPad Software), which was also used for statistical analyses. One on one comparisons of normal distributions were performed using unpaired *t*-tests. Xenograft growths with the different inhibitors were compared with two-way ANOVAs. In several panels all the datapoints per condition are provided. Alternatively, bar graphs illustrating the mean ± s.d. are provided.
